# Parental Perspectives on Family Mealtimes Related to Gastrostomy Tube Feeding in Children

**DOI:** 10.1177/1049732321997133

**Published:** 2021-03-05

**Authors:** Ellen Backman, Mats Granlund, Ann-Kristin Karlsson

**Affiliations:** 1 Halmstad University, Halmstad, Sweden; 2 Region Halland, Kungsbacka, Sweden; 3 Jönköping University, Jönköping, Sweden

**Keywords:** developmental disabilities, enteral nutrition, ecocultural theory, interviews, pediatrics, qualitative, stimulated recall, Sweden

## Abstract

Built on the important functions daily routines serve families and child health, this study aimed to explore parents’ descriptions of mealtimes and food-related challenges when living with a child using a gastrostomy feeding tube. The study was informed by ecocultural theory and based on in-depth interviews combined with stimulated recall. The interviews of 10 parents were inductively analyzed by means of qualitative content analysis. Four main categories comprised the parents’ descriptions: “One situation, different functions,” “On the child’s terms,” “Doing something to me,” and “An unpredictable pattern,” with one overarching theme. The analyses showed that the parents strived to establish mealtimes in line with their cultural context, although they struggled to reach a point of satisfaction. The study highlights the importance of health care professionals to address the medical aspects of caring for a child with a G-tube, but also the potential psychological and social consequences for ordinary family life.

Since the introduction in 1980, the placement of a gastrostomy tube (G-tube) has become the preferred feeding method for children with prolonged inadequate oral intake. A G-tube is a surgically placed device giving direct access to a person’s stomach for supplemental feeding, hydration, or medication (Fröhlich et al., 2009; Gauderer et al., 1980). The practice of G-tube feeding is indicated for children due to acquired or developmental disorders, most commonly neurological impairments, congenital malformations, or oncologic illness (Fröhlich et al., 2009; Glasson et al., 2018). The G-tube may be used to occasionally administer supplementary feeding as part of a nutrition support regimen or be the sole route of nutritional intake. Several studies have shown G-tube feeding in children to be a safe and effective method of nutrition support, resulting in weight gain and tolerable complication rates (Duncan et al., 2018; Lalanne et al., 2014; Wu et al., 2013).

Hubbard and Mayre-Chilton (2015) reported on the experiences of young adults with G-tube placement during childhood, describing that the participants felt more socially included after G-tube placement and experienced reduced stress during mealtimes for the whole family. However, previous research on parental experiences of pediatric G-tube use has revealed practical challenges and psychosocial issues. Brotherton and Abbott (2012) as well as Brotherton et al. (2007) conducted semi-structured interviews with parents to explore the decision-making process and the impact of G-tube feeding on daily life. Similar to the young adults, the parents reported reduced pressure at mealtimes and satisfaction with their child’s feeding regimen, but also a feeling of being forced to accept the G-tube insertion, problems with supportive childcare, and restricted ability for the parents to leave the home. Other daily parental challenges associated with pediatric G-tube feeding include practical limitations related to carrying and sterilizing equipment (Craig & Scambler, 2006), dilemmas relating to maintaining participation in everyday activities, managing responses to the use of tubes for feeding and doing what feels right for their child (Edwards & Leafman, 2019; Hopwood et al., 2020), caregiver burden handling health care contacts (Backman et al., 2020), and behavioral problems in children (Burklow et al., 2002). Russel et al. (2017) explored the family mealtime routines of six families of children with cerebral palsy who required G-tube feeding. The study participants reported difficulties integrating the tube feeding into the daily mealtime practice and a need for support when developing new routines and rituals to maintain a sense of normalcy in the family. The authors concluded that to enhance the success of home-based interventions such as G-tube feeding, more research is needed to explore parents’ abilities to adapt and strategize to these interventions. The present study set out to further explore how family mealtimes are organized when facing long-term health conditions and G-tube feeding as this type of knowledge would increase the understanding of parents’ roles and actions when adjusting to everyday life with pediatric G-tube feeding.

The theoretical approach is informed by ecocultural theory (Gallimore et al., 1993; Weisner, 2002), as this theory strives to explore and understand the factors influencing and shaping the family dynamics and sustainability of daily routines and rituals. The establishment and maintenance of daily activities, such as child-rearing practices and traditions, guide families’ conscious and unconscious tasks and behaviors as well as organize and shape children’s activities and development (Spagnola & Fiese, 2007; Weisner et al., 2005). Ecocultural theory emphasizes that the major task for all families is the ability to find meaning in routines and rituals and that families with the ability align family members’ interests, needs, competing goals, and resources with the family’s valued routines and rituals are more likely to experience well-being and support the optimal development of their child (Gallimore et al., 1993; Weisner et al., 2005). The theory has been proven valuable in deepening the understanding of factors that shape routines related to family mealtimes (Bekelman et al., 2019).

According to the Cambridge Dictionary (n.d.), a *routine* is a usual or fixed way of doing things, and a *ritual* is a set of fixed actions or sometimes words performed regularly, especially as part of a ceremony; these two concepts are thus similar in terms of recurrence but differ in their symbolic meaning. Routines and rituals can be contrasted along the dimensions of communication, commitment, and continuity (Fiese et al., 2002). Routines typically involve instrumental communication relating to what must be done during specific, reoccurring activities and involve a momentary time commitment. Rituals convey messages of identification and social inclusion through affective commitment in activities, providing continuity in meaning across generations (Fiese et al., 2002).

The family mealtime comprises routine segments, such as the instrumental setting of the table or preparing vegetables, along with symbolic components in the choice of food being served at specific events or reciting a blessing (Fiese et al., 2006). Mealtimes thus reflect more than a single situation, they reveal the way food is viewed in a family and offer opportunities to explore patterns of social interaction, the roles of individual family members, and the management of mealtime challenges as well as the deeper cultural meanings of the event (Fiese et al., 2006; Ochs & Shohet, 2006).

Spagnola and Fiese (2007) suggest that routines and rituals, for example during mealtimes, form a foundation which to return to in times of stress or change. However, previous research in families of children with developmental disorders suggests that routines and rituals can be difficult and stressful to maintain depending on the child’s specific health condition (Crespo et al., 2013; Santos et al., 2018). In ecocultural theory, accommodation is the concept used to explain families’ adaptive strategies and functional responses to the demands of daily life (Gallimore et al., 1989). Examples of accommodation areas are childcare tasks, social support, and domestic chores. The process of making conscious or unconscious accommodations leads to a daily life that is adapted to a family’s needs, beliefs, and values according to internal and external resources (Weisner, 2002). In a study on how parents learn to overcome challenges related to tube feeding, Hopwood et al. (2020) demonstrated four kinds of tools parents used to adapt: memory aids and readiness tools, metaphors and narratives, repurposed everyday objects, and personalized routines and materials. What is lacking from this, and other previous reviewed studies, is focused on the accommodations related to family mealtimes. Therefore, this study aimed to explore parents’ descriptions of family mealtimes and food-related challenges when living with a child using a G-tube by focusing on the concreteness of family mealtimes.

## Method

A qualitative descriptive design (Sandelowski, 2000) was applied in the study with a stimulated-recall interview methodology. Stimulated recall presents participants with an opportunity to discuss strategies or activities while they are directly presented with video-recorded examples of themselves engaging in interaction (Lyle, 2003). The methodology complements more commonplace interview procedures where participants are asked to remember actions that they have taken or the values and strategies that they generally use (Dempsey, 2010). Using visual methods, such as video recordings, in combination with other forms of data collection can elicit deep insights from participants and ensure clarity in questions and response, for example (Glegg, 2019). In the research context of childhood disability, Wilder (2008) reported that using video recordings of everyday interactions when interviewing parents complemented other research methods and added greater depth to the data material. Activity settings are the core unit of analysis for ecocultural theory and comprise five components. These components are the people present, cultural values and goals, the tasks being performed, scripts for conduct, and the participants’ motives and engagement in the activity (Gallimore et al., 1989). In this study, stimulated recall using recorded family mealtimes was chosen to enable conversation about this particular activity setting and the family’s consciously or unconsciously performed actions and strategies. The design of the study was piloted in one family so that the procedures could be refined for clarity and ease of use. The pilot interview was included in the present data analysis as no major changes were made.

### Participants and Research Context

Families were recruited from three administrative regions in Sweden, using the inclusion criteria of having a child aged 6 to 12 years with a G-tube activity exceeding 1 month. The focus was on children presented with clinical conditions that would widely be viewed as “long-term health conditions.” This included conditions such as congenital physical and/or intellectual disabilities deriving from diagnoses such as neurofibromatosis, cerebral palsy, malformations, or muscular dystrophy. Children with malignancy and traumatic brain injury were excluded. To reach maximum variation in the empirical material, purposive sampling was applied relating to sex, underlying diagnoses, time since G-tube placement, and amount of food taken by mouth (Sandelowski, 2000). The age range of the children was chosen to make individual interviews with the children possible. Seven families (six two-parent families and one single-parent family) were included, and interviews were conducted separately with parents and the children.

Parents of three girls and four boys with various health conditions participated. Specific diagnoses of the children are not reported to preserve the anonymity. There was a diverse range of feeding regimes, including children receiving all nutrition via G-tube, to children only occasionally using the G-tube for additional fluids or medication. Feeding was administered automatically using a pump for some children and manually using a syringe for others. Data from the child interviews have been analyzed and are reported separately.

The parents were given the option to be interviewed individually or together. A total of 10 parents were interviewed. In three interviews, the mother and the father were interviewed together, and in four interviews, only the mother was interviewed. All parents had Swedish as their first language and lived in towns and rural areas in the south of Sweden.

### Data Collection

An initial home visit was scheduled for the researcher to provide details of the study and to answer any questions. At this visit, the families were supplied with a small digital camcorder and were instructed to record three ordinary family mealtimes over the course of a 2-week period. Thus, the recordings were performed by the families themselves, thereby minimizing researcher interference. Families were instructed to start recording at what they considered to be the beginning of the mealtime and to stop recording when the mealtime was finished. The parents selected one of these recordings that they felt best captured their typical family mealtime and that they felt comfortable sharing with the researcher. The interviews with the parents were scheduled to allow the researcher to look through the recording in advance (time between recorded mealtime to interview ranging from 2 to 48 days, median 14 days). In all cases, recordings started with family members gathering around the table to finalize mealtime preparations and ended when all members had left the table. The recordings were reviewed using an observation template organized according to the five components of an activity setting within ecocultural theory. The observation template assisted the researchers to keep the focus on the study aim across the different families’ recordings and structure the viewing. Sequences that evoked questions related to accommodations, routines, and rituals of the mealtime were selected to present at the coming interview.

The interviews were scheduled at a location and time of the parents’ choosing to ensure an optimal interview situation (Kvale, 2007). The majority opted to hold the interview in their own home, whereas two interviews were conducted at the local hospital. The knowledge among the authors in family-centered health care was essential in how the data collection was organized and executed. For example, this meant to be flexible and open to any desires of individual families to facilitate participation.

The interviews began with asking the parents about their day-to-day lives in relation to food, eating, and mealtimes, without watching the recording. An interview guide composed of open-ended questions directed the interview toward ecological features (e.g., context, domestic chores, social support), values and goals (e.g., the meaning of and commitment in activities), and personal characteristics (e.g., health conditions and competencies of family members) (Gallimore et al., 1989) and was used to explore the parents’ descriptions and experiences of former as well as present family mealtimes (Supplemental Table 1). To capture the dimensional facets of mealtimes, a mealtime was defined in line with Larson et al. (2006) as a coordinated series of activities, including shopping or gathering food, meal preparation, eating, social interaction, and cleaning up. Following these initial questions, the selected sequences from the recorded mealtime were presented to the parents on a laptop. The parents were encouraged to respond freely to topics introduced by the researcher. One example from an interview was, “In this sequence, you pretend to eat the child’s food. Tell me more about that.” As shown in this excerpt, the parents were encouraged to reflect upon the actions during the specific mealtime. Furthermore, the video sequences gave the parents an opportunity to relate what was seen in the recording with their previous accounts. The parents were also invited to stop the recording at any time and introduce areas for reflection. Although recordings mostly focused on eating and social interaction, all dimensions of the mealtime definition were covered in the interviews.

In two interviews, the parents felt uneasy watching themselves, and the recorded mealtime and selected sequences were instead described in words by the researcher as a basis for the discussion. The interviews were carried out between January 2019 and December 2019 and were audiotaped using a Sony mp3 IC recorder. The interviews lasted between 42 and 111 minutes (median, 77 minutes) of which 27 to 50 minutes (median, 36 minutes) relied on stimulated recall.

### Data Analysis

The video recordings of family mealtimes yielded visual data, but the verbal material from the parents’ explanations and descriptions of the specific mealtime, taken together with the conversations guided by the thematic questions, provided the data for analysis. The audio recordings were transcribed verbatim. The transcribed text was subjected to qualitative content analysis with an inductive approach, which was conducted in several predefined steps (Graneheim et al., 2017; Graneheim & Lundman, 2004; Lindgren et al., 2020). Qualitative content analysis was chosen as it uses well-defined tools for the phenomenological description of the manifest, concrete content as well as hermeneutic interpretations of the latent abstracted message within the participants’ experiences.

In the initial step of the analysis, the full transcripts were read to get a sense of the whole. Next, meaning units relating to family mealtimes and food-related challenges were identified and condensed with the core meaning preserved. To prevent fragmentation, a meaning unit could vary between a short sentence to longer statements that related to each other through their content and context. The condensed text was coded, sorted, and abstracted into 17 subcategories and four main categories (Supplemental Table 2). This process was iterative, moving back and forth between the different steps in the analysis and reflected the manifest content of the interviews. The NVivo 1.3 software package (QSR International, 2020) was used to organize and aid the analysis in combination with manual sorting. Finally, an overarching theme was interpreted, corresponding to the latent content of the interviews. The tentative categories and theme were considered and discussed in the multidisciplinary research group. Differences emerged in relation to the approach to interpreting accounts of health care contacts, school adjustments, and support from friends and relatives. Differences were resolved through a process of further analysis and reflective discussions.

### Ethics

Ethical approval was obtained from the responsible regional ethical review board. Written informed consent to participate was obtained from the parents prior to the filming and interviews. In accordance with the Helsinki Declaration (World Medical Association, 2013), the consent form provided assurances that participation in the study was completely voluntary and that the confidentiality of the families would be protected. Parents were also fully informed about their right to withdraw at any time without consequences, how the collected material would be kept, and their right to receive information about stored personal data following the General Data Protection Regulation (GDPR; European Union, 2018).

## Findings

The 10 interviewed parents of children with a G-tube described their family mealtimes and food-related challenges as “One situation, different functions,” “On the child’s terms,” “Doing something to me,” and “An unpredictable pattern,” which was interpreted as mealtimes being “An ever-changing kaleidoscopic experience” (Table 1). The categories are reported in the following headings with subsequent subcategories and illustrated with quotations from the original interviews.

**Table 1.** Overview of Subcategories, Main Categories, and the Overarching Theme.

Subcategory	Category	Theme
A moment of togetherness	One situation, different functions	An ever-changing kaleidoscopic experience
But one of many daily musts		
Ensure bodily needs		
Improve the child’s eating		
Arrange the setting	On the child’s terms	
Comply with food preferences		
With regard to physical state		
Respect the child		
Always on call	Doing something to me	
Being the unusual one		
Having self-trust		
The healthcare system and us		
Touch on the future		
The child during mealtimes	An unpredictable pattern	
Practicalities		
Foods of tradition		
Our family’s interaction		

### An Ever-Changing Kaleidoscopic Experience

At the core of the parents’ narratives was how their descriptions moved between the deepest sorrow to sheer happiness and between mundane practicalities to philosophical reflections on meanings and dilemmas. This was interpreted into the overarching theme of mealtimes as “An ever-changing kaleidoscopic experience,” capturing how mealtimes were assembled from smaller parts that were not always of interest on their own, but taken together, created a fascinating, complex representation. Another aspect of the kaleidoscopic metaphor was how the slightest shift in perspective could change the picture entirely even though the basic components remained the same. Furthermore, the kaleidoscopic metaphor reflected how all experiences, good or bad, happy or sad, together created a unique, individual image that described the reality for a particular family.

### Main Category 1: One Situation, Different Functions

This main category rests upon the four subcategories “A moment of togetherness,” “But one of many daily musts,” “Ensure bodily needs,” and “Improve the child’s eating.” The category echoed the different functions the family mealtime carried for the parents, ranging from a valuable opportunity to participate in each other’s daily lives, regardless of the amount of food that was actually consumed, to an activity needed to satisfy the nutritional needs of the individual child with the G-tube. The parents’ accounts reflected a situation where mealtimes meant navigating between these functions and striving to keep mealtime a positive experience for all family members while making it as beneficial as possible for the child with a G-tube. In the subcategory “A moment of togetherness,” all parents described mealtimes, especially dinnertime, as a cherished social occasion, an opportunity to gather the family to cook together in the kitchen or sit down around a shared table. One father explained how they adjusted dinnertime to fit into the schedule of the five family members:

Our children have a lot of activities, but we always try to sit down together, all of us to eat, even if it’s late in the evening, . . . but then we’ll all be sitting there and it’s a family meal.

In this example, the father described a compromise between what he saw as the optimal dinnertime and the ability to share the event with all family members. For the family, eating together was more important, thus the dinnertime was a secondary consideration. When one mother was asked to clarify why she perceived mealtimes as important, she explained, “It’s important that you sit down together and talk, I think, we’ve always done that, at least once a day.” This excerpt highlights not only the importance that sitting down together had for the family, but also that a daily shared mealtime was deeply rooted in family traditions, where the collective experience of sitting down to dinner was highly valued.

On the contrary, the mealtime was also conveyed by the parents as being “But one of many daily musts” and therefore needed to be limited in terms of the time and effort expended in relation to other daily tasks. The subcategory “Ensure bodily needs” comprised descriptions of how the parents tackled the task of getting the child to eat as much as possible without mealtimes becoming overly negative or demanding for the child and the rest of the family. For the children who used the G-tube only occasionally, mealtimes were imperative for ensuring all of the child’s nutritional needs were met, and these parents described how they juggled numerous strategies to promote the child’s oral intake while they tried to eat themselves. The parents of children where the G-tube feeding provided all the nutrition the child needed reported less stress in relation to the nutritional content of the food.

The last subcategory, “Improve the child’s eating,” collected the parents’ accounts of mealtimes as an opportunity to challenge and stimulate the child’s specific abilities to manage food intake. One mother described how she tried to use questions about the food during dinnertime: “It’s a way of getting him to approach food a little . . . like ‘How does it smell?’ ‘How does it feel; is it warm or cold?’ ‘Is it hard or soft?’ . . . we try to talk about that.” As illustrated, she purposefully encouraged her son to explore various aspects of the food, not by having him taste or eat, but by verbalizing sensory aspects to evoke curiosity about the food. Other approaches included routinely serving the child a portion of what the rest of the family was eating or including the child in setting the table.

### Main Category 2: On the Child’s Terms

This main category was developed from the parents’ descriptions of how mealtimes were focused around the child’s specific needs and resources, with other family members’ needs sometimes being secondary. For some parents, this meant going against their own principles of what was accepted during mealtimes or having different approaches toward the child compared to siblings. The category was built upon the four subcategories: “Arrange the setting,” “Comply with food preferences,” “With regard to physical state,” and “Respect the child.” The parents’ descriptions illustrated accommodations that were practical in nature, relating to the physical environment, and accommodations to the parents’ own attitudes toward the child. Within the subcategory “Arrange the setting,” examples were reported where the child was allowed to watch videos on a tablet during mealtimes to maintain interest in being present at the table or planning seating arrangements according to the child’s requests, although these were not opportunities available to other family members. Parents also reported being conscious in their choice of conversation topics and how they approached the child with questions or tasks to set the child up for success during mealtimes in terms of the intake of food.

The subcategory “Comply with food preferences” depicted how the parents served food types and food amounts that they knew their child favored. For several parents, this interfered with how they ideally would want to plan the family’s food, resulting in less variety and experimental cooking than would have been the case in the absence of the child’s specific needs. Furthermore, the parents gave accounts of being conscious of the child’s bodily condition when arranging family mealtimes. These accounts comprised the subcategory “With regard to physical state” and were often related to balancing the amount and pace of enteral feeding with food taken orally to prevent nausea and vomiting. The parents also expressed how it was essential to be responsive to the child’s chewing and swallowing abilities and general health status so that they could make appropriate demands and provide an adequate level of support.

The subcategory “Respect the child” captured descriptions of how the children were given space to be independent and make their own mealtime decisions and how children were offered their own portion of food even if they only ate a negligible amount. One mother provided an example of a family tradition relating to food: “When it’s your birthday, you get to decide on food . . ., which she’s fully aware of and does.” As this excerpt shows, this family’s custom of deciding what food to eat on one’s birthday was consistent, even for a child who did not eat large amounts orally. Respecting the child could also involve allowing the child to order something for themselves at a restaurant, yet accepting the fact that the child might leave most of the meal on the plate due to a lack of appetite. Another example was given by a mother, who recounted how food was served to her child and other children at family gatherings, “It is really important that, even if he knows he won’t eat it all, he still gets the same as the rest. . . . he shouldn’t be treated differently.” This report shows that the mother was aware of other meanings coupled with food beyond actually eating it, such as the significance of her child not being singled out as different in relation to other children.

### Main Category 3: Doing Something to Me

Parents of children with a G-tube described mealtimes in terms of how the experience affected them as a person. The category encompassed the subcategories “Always on call,” “Being the unusual one,” “Having self-trust,” “The health care system and us,” and “Touch on the future.” The child’s eating difficulties and the use of a G-tube meant that the parents always needed to be prepared to respond to situations that may arise before, during, or after mealtimes. Being “Always on call” involved staying a step ahead to make certain that the child’s preferred dishes were always on hand, maintaining daily contact with the school to be updated on food intake or arranging medical certificates that made it possible, for example, to bring the child’s enteral feeding supplies when traveling. In addition, the parents had to manage how the child’s different eating routines affected them and the spectrum of emotions that evoked.

The subcategory “Being the unusual one” delineated accounts of anxiety preceding the insertion of a G-tube, handling others’ negative attitudes and questioning one’s own abilities as a parent, but also accounts of the joy and feelings of security related to the G-tube and the progress in the child’s eating. Although the parents recalled feeling reluctant and anxious about the G-tube before insertion, the G-tube was now seen as a source of relief, both by relieving pressure related to ensuring the child gets adequate nutrition at mealtimes and by replacing the nasogastric tube, which several children in the study had taped to their faces, with a skin-level device that could be hidden under the child’s clothing. Another positive aspect was how the G-tube signaled to others that the child’s eating was “a real problem,” not something that resulted from parental failure. Several parents described how it was difficult for others to understand the child’s impairment. One mother responded to a question about the origin of her son’s feeding difficulties, saying, “I don’t know, he’s never liked food, he’s never eaten, [sighs] . . . but relatives and friends think that you as a parent are doing something wrong, that it is our fault that he doesn’t eat.” In this example, the mother shed light on how she must deal with both her own understanding of her child’s feeding difficulties and others’ perceptions of her as an incapable parent. Family mealtimes outside of the home were also situations where parents constantly had to negotiate with themselves, balancing between letting the child eat in his or her preferred way and exposing parents or siblings to the glances or opinions of others.

The subcategory “Having self-trust” comprised accounts of how parents had found their own solutions to make everyday life work in the absence of external support and to rely on their own abilities. One example was a boy who had used the G-tube when he was younger, but now used it only sporadically. His mother expressed: “I don’t think he needs nutritional supplement anymore. Not that I have done any analysis of whether he gets what he needs, but in any case, he looks happy and energetic, I think it works.” The example highlights how this mother had confidence in her ability as a parent to look out for her child’s best interest based on what she observed in daily life, without the need for external investigations.

The subcategory “The health care system and us” covered how parents experienced health care services relating to the child’s eating and family mealtimes. For some participants, health care professionals provided valuable support, reinforcing parents in their role and allowing the family to set the pace of health care measures while providing external expertise in treatment options. For other families, the support received from health care services was perceived as disorganized and unsatisfactory, for example, requiring the family to be sent between different clinics, recommending unrealistic treatment options or simply providing no treatment at all. One mother exclaimed, “The health care services don’t always have anything more than medical knowledge. They just think ‘gastrostomy button and function,’ not ‘daily life, family and getting it to really work,’ you know.” As the statement by this mother shows, she was dissatisfied with the absence of a family perspective in the care of her child’s G-tube and the lack of insight among health care professionals for how the insertion of a G-tube could affect the child and family beyond the medical aspects. Several parents perceived that health care services “brought out the worst in them” and that all they did was complain in an attempt to get the support they needed for their child and themselves.

In the parents’ accounts, some dared to “Touch on the future,” reflecting on not only thoughts of the near future but also further on in the course of time. In terms of the near future, parents expressed feelings of inadequacy in terms of how to best support the child’s eating development and the tiring struggle to ensure the best possible mealtime assistance at school and through health care services. Thinking further ahead, some parents thought the child would always require nutritional support in one way or another, expressing concerns of how the G-tube would affect the child when he or she grows older. Others spoke confidently about how the child would manage to eat without the G-tube in years to come. One mother voiced her vision for the future, saying, “The dream is of course that our child will sit with us, being able to eat, maybe his own food, but that he sits with us and eats when we eat.” Her account elucidated the tremendous value of the family mealtime, that it was something worth dreaming about. She was not alone in this view; several other parents shared a future vision of being able to have “just an ordinary family meal.”

### Main Category 4: An Unpredictable Pattern

The parents’ mealtime experience as “An unpredictable pattern” was derived from the subcategories “The child during mealtimes,” “Practicalities,” “Foods of tradition,” and “Our family’s interaction” and reflected how mealtimes were realized in the family. The descriptions depicted mealtimes as having clear borders and recurring elements relating to structure, type of meal, content, and people’s actions. However, at the same time, the parents talked about never being sure how the next mealtime would develop given the uncertainty about what the child with the G-tube was going to eat or how long it would take, something that tested the whole family. Another aspect of mealtime unpredictability was voiced by one mother: “We don’t have many fixed habits really, but it feels pretty nice, because then you can relax.” Her account showed that intentionally casting routines aside was something positive and could be a way for parents to feel more free.

The subcategory “The child during mealtimes” comprised accounts of how the children participated in food-related activities and how the parents perceived their children’s interest in mealtimes. Many parents described an apparent improvement in family mealtimes stemming from the child’s acceptance of sitting at the table together with the rest of the family, increased tolerance for different types of foods, and the fact that the child now contributed to conversations at the table to a larger extent. However, there were also examples of parents who reported that their child had little curiosity about food and expressed a feeling of frustration over the time allocated to eating. Family mealtimes were described as full of “Practicalities,” including scheduling, seating arrangements, people present, and assigned roles during mealtimes. Within this subcategory, parents also mentioned demands to meet food and time requirements in relation to allergies or diabetes as well as establishing functional rules of conduct within the family.

The subcategory “Foods of tradition” sheds light on descriptions of food and mealtime habits associated with special weekdays or more traditional events, such as Christmas and Midsummer celebrations. The parents gave examples of food-related traditions founded on the cultural and social Swedish context in addition to traditions that parents had created or adapted based on the child’s abilities and interests. In some families, traditional dishes were served alongside other dishes to ascertain that there would be something to eat for everybody. One mother vividly described the family’s Christmas preparations and explained, “we never buy Christmas treats, no, we make it ourselves, and she takes part in doing it, then we have one of those cozy days, just standing there, baking.” Her report communicated how the tradition of making Christmas treats was perceived as important both for her as a parent and her child with a G-tube. On the contrary, she also reflected on the complications associated with maintaining food-related traditions:

It is . . . not possible when you have a child like that . . . I mean, if she says she’s in the mood for tacos on a Monday, then you can’t say “No, not today, because we eat tacos on Fridays.” No, but “okay” you say, “then we will have tacos.” You have to adjust to what she is in the mood for.

These two accounts exemplify how the mother negotiated between the value of traditions, that is, having special dishes to look forward to, and the need to get her child to eat as much as possible.

The last subcategory, “Our family’s interaction,” covered descriptions of social interaction during mealtimes. The parents depicted family mealtimes as characterized by a range of communication features. Some of these were anchored in the present activity and included remarks about behavior, verbal prompts to support nutritional intake, and comments on the mealtime task itself. Other communication features included topics outside the immediate mealtime context: the planning of future holidays, recollection of past events or conversations about news, and other happenings in the world. A feeling of strained interaction was common among the parents, and it was perceived that this primarily derived from the number of reminders given to the child with the G-tube but also from tension between siblings or parents’ workload. One father described dinner as “the most boring meal of the whole day because I don’t have any positive associations with it anymore, because of all the squabbling and coaxing.” His statement reflected how the accumulation of prior strained interaction patterns continued to affect current interactions at mealtimes. This can be contrasted against his previously referenced comment in the main category “One situation, different functions,” where he emphasized the value of shared family meals. The significance of family mealtimes as an arena for communication was evident in several of the parents’ accounts, pointing to dinners specifically as a treasured event because of the opportunity for small talk, jokes, the exchange of information, or just sitting quietly together. Thus, the parents’ experiences also touch on the emotional realm to a large extent and how family communication during mealtimes created a sense of belonging and shaped the family identity.

## Discussion

The aim of this study was to explore how parents described family mealtimes and food-related challenges when living with a child using a G-tube, informed by ecocultural theory. The overall finding of this study was that the experience of family mealtimes for families of a child with a G-tube is multifaceted and includes several interrelated, but also conflicting dimensions. The mealtime experience was, on one hand, closely related to the individual needs, abilities, and behaviors of the child with a G-tube, but on the other hand, was referred to as a collective event, essential for social interaction and the creation of family identity. This major finding suggests that parents constantly balance between an individual or collective responsibility. A dimensional understanding of the family mealtime dictates that these two positions, the individual and the collective, cannot be easily divided, yet may take up differing amounts of space and energy for families depending on external support, internal resources, and present demands. Also, the integrative feature of these two positions sheds light on the need for health care services to offer support in areas of family communication, mealtime routines and rituals, founded in the family’s cultural context, not only in medical areas relating to G-tube maintenance and growth follow-up. During the child’s life, much time and effort had been assigned to hospital visits, feeding arrangements, and medical care according to the parents. But despite the fact that the child’s feeding difficulties had marked both the former and present family life, parents expressed a lack of interest in health care professionals to gain an understanding or provide support beyond the practical handling of the G-tube or the nutritional intake of the child. The importance of a more multidimensional approach health care has been put forward by Craig (2013) and Backman et al. (2020) as well as in the assessment framework suggested by Brotherton et al. (2007). The findings from this study expand upon previous research by focusing on the ecocultural context of the child. If health care measures are to be meaningful, support must be tailored to meet the goals and values of the specific family.

Family mealtimes have been called “densely packed events” that shape child and family well-being by covering practical aspects of food consumption, role assignment, making plans, and reviewing past events (Fiese et al., 2006). The family mealtime in this study was experienced as having several functions. These included ensuring bodily needs and improving the eating abilities of the child as well as the value of family members gathering around the table and serving specific dishes, irrespective of whether any food was actually consumed. Consistent with earlier findings by Crespo et al. (2013) and Santos et al. (2018), mealtime routines and rituals were reported to be valuable and desirable, yet difficult to establish at times due to the unpredictable nature of the child’s eating. Spagnola and Fiese (2007) suggested that routines and rituals form a foundation that families can return to in times of stress or change. The experiences reported in this study showed that parents of children with G-tubes found comfort in and strived to establish mealtime routines and rituals in line with their social and cultural context, although they struggled to reach a point of satisfaction. In some instances, parents adjusted routines and rituals to better suit the child with a G-tube, in other cases, parents focused on the wishes of the rest of the family.

Fiese et al. (2006) concluded that communication patterns in families constitute an essential ingredient in understanding the benefits and challenges of mealtimes. Clear and direct communication cultivates an environment of emotional support and makes it easier to get mealtime-related tasks done. For the parents in this study, communicative features during mealtimes were used both as tools to develop abilities and to prevent problems. Although seen as troublesome in some cases, the parents’ accounts highlighted communication as something that defined their family and socially constructed their relationships. This study was not designed to explore linguistic features or communicative patterns within the mealtimes of the participating families. Future studies in this area could focus on expanding the knowledge of more specific communicative aspects to gain an understanding of how mealtime dynamics influence both eating and communicative development in children with G-tubes.

In terms of ecocultural theory, the findings depicted how the health condition of the child and the food-related challenges had a negative impact for many of the families, but also depicted the accommodations made to approach these challenges. Consistent with this study’s focus on family mealtimes, accommodations related to domestic chores and tasks were most evident, such as scheduling mealtimes according to the child’s enteral feeding scheme, planning family dinners according to the child’s food preferences, and avoiding certain conversation topics to prevent tantrums that could interfere with the child’s food intake. However, in terms of family mealtimes, parents also gave examples of accommodations in childcare tasks, marital roles, and health care services, mirroring previously described accommodations in by families of children with chronic health conditions (Crespo et al., 2013; McConnell & Savage, 2017). One area of accommodation often mentioned by the parents in this study was social support. In line with Brotherton et al. (2007), who also interviewed parents of children with G-tube feeding, parent accounts of difficulties going out as a complete family, rarely having contact with relatives, and reduced opportunities for parents to spend time together were recurring themes. In this study, the lack of support from relatives and friends caused families to arrange their everyday lives to manage on their own. Many parents sought out digital communities as a way to feel less alone and to get advice from others in similar circumstances. Based on parent interviews, Brotherton et al. (2007) indicated that the combination of the G-tube feeding and the child’s underlying disability created a division within the immediate family as well as the extended family. Although this also seemed true for many of the parents in this study, it was contrasted with accounts of accommodations made to promote family cohesion during mealtimes and expressions of how the G-tube had supported the ability to share family experiences without the stress of ensuring adequate nutrition received orally. In line with previous research (e.g., Wilder & Granlund, 2015) on accommodations in families of children with disabilities, the present findings suggest that accommodation cannot be seen as a single process exclusively centered around the child with a G-tube. Rather, multiple intertwined and changing accommodation processes were evident in the parents’ accounts, driven by various motivational forces in different families. The activity setting is the core unit of analysis in ecocultural theory and provided a foundation to understand what lie behind families’ accommodations. One example was how mealtime scheduling was adjusted according to the needs of a family member with diabetes. Another example was the arrangement of childcare tasks with siblings being the primary focus and the child with the G-tube being the secondary focus. These findings can be understood in terms of the hierarchical nature of accommodations (Gallimore et al., 1989) and the fact that depending on the specific family, some accommodations are more valued and given higher priorities than others.

Craig and Scambler (2006) suggested that having a child with a G-tube meant that parents needed to negotiate between the idealized picture of parenthood, that is, what being a parent should be like, and being the parent of a child who needs a G-tube and the stigma that goes along with it. This negotiation was also noticeable among the parents in this study. It involved being the expert on one’s own child and home environment while being guided by and in the hands of the experts in the health care services. It involved bearing the ultimate responsibility for the health of your child yet perceiving that others assign you an unspoken sense of accountability for the child’s feeding difficulties. Furthermore, it involved being happy for and loving your child, yet feeling ashamed and concealing the child’s feeding and eating differences. For some of the parents in the current sample, a strong sense of self-trust and robust collaboration between parents had steered them toward a position where they now relied on their own way of managing daily struggles and a feeling of resignation pertaining to life areas beyond their control. Other parents revealed feelings of vulnerability, loneliness, and abandonment related to family mealtimes and their child with a G-tube. Previous researches have described that parents of children with G-tube feeding face dilemmas concerning their parental obligations toward the child with disabilities, that is, searching for and providing the best possible care and prioritizing time to support the child, contrasted against the needs of siblings and “normal parenting” (Hopwood et al., 2020; McConnell & Savage, 2017). McConnell and Savage (2017) suggested that family life to a large extent revolved around the child with disabilities and that parents were caught in a “moral bind.” The parents of this study expressed similar dilemmas concerning siblings. In addition, the parents expressed an internal negotiation relating to family values and rules of conduct. What was best for the child’s nutritional intake was not always consistent with the parental view of what encompassed a “good family meal.”

### Limitations and Directions for Future Research

The main strength of this study was the qualitative approach, which used in-depth interviews together with video recordings from the participating families. By using a method combining the opportunity for parents to recall and describe general family mealtime practices with reflections on a specific recorded event, an incredible amount of rich material was produced from what may be considered a limited number of interviews. Thus, the 10 participants were assessed to be sufficient, as trustworthiness in a qualitative study is gained more by the richness of each interview than by sample size (Sandelowski, 2000). Another strength was the participation of both mothers and fathers, as previous studies have been dominated by a maternal perspective (Adams et al., 1999; Craig & Scambler, 2006; Wilken, 2012). However, certain limitations also need be considered. Video recordings are fractional, capturing only part of an event, depending on where the camera is directed, for how long and how it is operated. Furthermore, video recording only reflects that specific mealtime, which might be different from the family’s usual mealtime. Another limitation with using video recordings is the possibility that the camera will affect family members’ natural behavior and how they interact with one another. Therefore, each family was asked to collect three recordings and choose the recording they felt best reflected their typical family mealtime. For future studies, it would be advantageous to include a greater number of recordings and/or other forms of data collection, for example, diaries or photos, to obtain a broader picture of the family mealtime. A limitation with the seemingly small sample size of families with children aged 6 to 12 years may be that the experiences of the interviewed parents only reflect those families that have reached a point in their daily lives where participation in research feels manageable. Thus, important information about children and parents who are still new to the routines and rituals of everyday life with a G-tube or about families who experience a significant degree of daily medical challenges may not be represented in this study. Studies including parents of younger children with newly inserted G-tubes may therefore be valuable in the future, as would studies exploring changes in accommodations over time for families of children with G-tubes.

## Conclusion and Clinical Implications

The ecocultural theory has a primary focus on the details of the day and how these shape parents’ experiences. The findings of this study suggest that the experience of family mealtimes is kaleidoscopic in families having a child with a G-tube and compels parents to constantly balance between individual or collective responsibility. A dimensional understanding of the family mealtime dictates that these two responsibilities cannot be easily separated, resulting in parents making daily accommodations to create a sustainable family mealtime. The findings call for external support for parents to prevent a feeling of seclusion and to aid in the establishment of everyday routines that align with family goals and values. Health care professionals can contribute to the delivery of health care that is built on shared power and collaboration by simply asking parents to tell their story and really listening to them. One recommendation for clinical practice would be to more regularly ask parents how they manage family mealtimes and what aspects they struggle with at home to tailor intervention strategies accordingly. Furthermore, both clinicians and researchers need to be aware that in addition to the challenges these families face due to their child’s health condition, they also face the same challenges as any other family in terms of a hectic work schedule, domestic chores, organized leisure activities, pets, and school assignments. When creating space for parents to talk about their everyday lives and what is important for them, one’s attention needs to be broadened to not only include the medical aspects of caring for a child with a G-tube, but also the potential psychological and social consequences for ordinary family life.

## Supplemental Material

sj-pdf-1-qhr-10.1177_1049732321997133 – Supplemental material for Parental Perspectives on Family Mealtimes Related to Gastrostomy Tube Feeding in Children Click here for additional data file. Supplemental material, sj-pdf-1-qhr-10.1177_1049732321997133 for Parental Perspectives on Family Mealtimes Related to Gastrostomy Tube Feeding in Children by Ellen Backman, Mats Granlund and Ann-Kristin Karlsson in Qualitative Health Research

sj-pdf-2-qhr-10.1177_1049732321997133 – Supplemental material for Parental Perspectives on Family Mealtimes Related to Gastrostomy Tube Feeding in Children Click here for additional data file. Supplemental material, sj-pdf-2-qhr-10.1177_1049732321997133 for Parental Perspectives on Family Mealtimes Related to Gastrostomy Tube Feeding in Children by Ellen Backman, Mats Granlund and Ann-Kristin Karlsson in Qualitative Health Research
