# Supplementary material for: Parental Perspectives on Family Mealtimes Related to Gastrostomy Tube Feeding in Children
Source: Qual Health Res. 2021 Mar 5;31(9):1596–608. doi: 10.1177/1049732321997133 (PMC8438777; doi:10.1177/1049732321997133)
Supplement: sj-pdf-1-qhr-10.1177_1049732321997133 – Supplemental material for Parental Perspectives on Family Mealtimes Related to Gastrostomy Tube Feeding in Children [file sj-pdf-1-qhr-10.1177_1049732321997133.pdf]

Supplementary Table 1.

Parental interview guide

**Questions without video**<br>*(follow-up questions to explore the where, who, what, how, feelings)*<br>1. Tell me about the mealtimes in general for your family.<br>2. Tell me about a meal you had in the family that you experienced as particularly successful or enjoyable.<br>3. At times, all families experience pressure and stress over different things. Tell me about a meal that you remember as affected by stress or that you experienced as less successful?<br>4. Families vary in what special habits and traditions related to food they have. Tell me how it is for you.<br>5. Tell me about the food and mealtimes for [your child with gastrostomy].<br>6. What are your thoughts about your child's eating in the future?<br>7. What support have you received related to your child's eating: from family, friends, or healthcare professionals?
**Questions with video**<br>*(follow-up questions to explore the where, who, what, how, feelings)*<br>1. Start by telling me why you chose this particular recording, in what way is this a typical mealtime for your family?<br>2. (Present examples of sequences, such as the participants' different roles and tasks, topics of conversation, interaction, adjustments during the meal, presence and involvement of the child with gastrostomy, activities).
**Closing the interview**<br>1. How did the camera affect your mealtime?<br>2. How did you feel about looking at your recorded mealtime and discuss it?<br>3. Now we've talked and looked at your recorded mealtime. I have no more questions. Is there anything else in your situation around eating and eating that you want to talk about?
