# Supplementary material for: Parental Perspectives on Family Mealtimes Related to Gastrostomy Tube Feeding in Children
Source: Qual Health Res. 2021 Mar 5;31(9):1596–608. doi: 10.1177/1049732321997133 (PMC8438777; doi:10.1177/1049732321997133)
Supplement: sj-pdf-2-qhr-10.1177_1049732321997133 – Supplemental material for Parental Perspectives on Family Mealtimes Related to Gastrostomy Tube Feeding in Children [file sj-pdf-2-qhr-10.1177_1049732321997133.pdf]

Supplementary Table 2.

Examples of meaning units, condensed text, codes, subcategories and categories.

Meaning unit	Condensed Meaning Unit	Code	Subcategory	Category
"The main thing is what's in his glass, he has to drink that [the nutritional supplement], he won't get away with less than that" (Gustav's father)	Won't get away with less than what's in his glass	Nutritional supplements	Ensure bodily needs	One situation, different functions
"Mixing different food doesn't work. She must have it in separate heaps on the plate. She must know exactly what there is" (Lisa's mother)	Mixed food doesn't work, needs to be served separately	Food serving	Food preferences	On the child's terms
"That feeling when I found the first Facebook group and just, 'There are others, it's not just us!' There are other parents going through the same thing, there are other children" (Karl's mother)	Through Facebook finding other parents and children going through the same thing	Not just us	Being the unusual one	Doing something to me
"There are times when she just eats a few bites, but other times when she gulps down a whole plate" (Alice's father)	At times, just a few bites, other times eats a lot	Amount of food	The child during mealtimes	An unpredictable pattern
